# Cross-Border Dissemination of Methicillin-Resistant *Staphylococcus aureus*, Euregio Meuse-Rhin Region

**DOI:** 10.3201/eid1505.071618

**Published:** 2009-05

**Authors:** Ruud H. Deurenberg, Eric Nulens, Havard Valvatne, Silvie Sebastian, Christel Driessen, Jos Craeghs, Els De Brauwer, Bernhard Heising, Yvette J. Kraat, Joachim Riebe, Frans S. Stals, Thera A. Trienekens, Jacques Scheres, Alexander W. Friedrich, Frank H. van Tiel, Patrick S. Beisser, Ellen E. Stobberingh

**Affiliations:** University Hospital Maastricht, Maastricht, the Netherlands (R.H. Deurenberg, H. Valvatne, S. Sebastian, C. Driessen, J. Scheres, F.H. van Tiel, P.S. Beisser, E.E. Stobberingh); General Hospital Sint-Jan, Brugge, Belgium (E. Nulens); General Hospital Vesalius, Tongeren, Belgium (J. Craeghs); Atrium Medical Center, Heerlen, the Netherlands (E. De Brauwer); General Hospital Düren, Düren, Germany (B. Heising); Orbis Medical and Care Center, Sittard, the Netherlands (Y.J. Kraat); Laboratory Aachen, Aachen, Germany (J. Riebe); Laurentius Hospital, Roermond, the Netherlands (F.S. Stals); VieCuri Medical Center, Venlo, the Netherlands (T.A. Trienekens); University of Münster, Münster, Germany (A.W. Friedrich)

**Keywords:** Antimicrobial resistance, staphylococci, cross-border healthcare, MRSA, Euregio Meuse-Rhin, MLST, PVL, SCCmec, spa typing, research

## Abstract

Because the prevalence of methicillin-resistant *Staphylococcus aureus* (MRSA) differs among the 3 countries forming the Euregio Meuse-Rhin (EMR) region (Belgium, Germany, and the Netherlands), cross-border healthcare requires information about the spread of MRSA in the EMR. We investigated the emergence, dissemination, and diversity of MRSA clones in the EMR by using several typing methods. MRSA associated with clonal complexes 5, 8, 30, and 45 was disseminated throughout the EMR. Dutch isolates, mainly associated with sequence types (ST) ST5-MRSA-II, ST5-MRSA-IV, ST8-MRSA-IV, and ST45-MSRA-IV had a more diverse genetic background than the isolates from Belgium and Germany, associated with ST45-MRSA-IV and ST5-MRSA-II, respectively. MRSA associated with pigs (ST398-MRSA-IV/V) was found in the Dutch area of the EMR. Five percent of the MRSA isolates harbored Panton-Valentine leukocidin and were classified as community-associated MRSA associated with ST1, 8, 30, 80, and 89.

## Abstract 2

MRSA clones were associated with hospital-associated clonal complexes and with Panton-Valentine leukocidin–positive community-associated MRSA.

Almost one third of the European population lives in a border region (Euregio). These border regions have collaborated since the late 1950s, especially in the field of healthcare (*1*). Cross-border patient mobility and free access to healthcare facilities within the European Union in general, and the Euregios in particular, are important for patients, medical doctors, healthcare facilities, and healthcare insurance companies. The Euregio Meuse-Rhin (EMR), an area totaling 4,973 square miles (12,882 km^2^), is the border region of Belgium, Germany, and the Netherlands. The EMR comprises the Belgian provinces of Limburg and Liège, the German-speaking region of Belgium, the Aachen region in Germany, and the southern part of the Dutch province of Limburg. Each year, thousands of the 3.88 million inhabitants of the EMR cross the border to consult a medical specialist or a healthcare facility. Since 2003, hospitals in the EMR have built a strong collaboration. For example, the University Hospital Maastricht in the Netherlands and the University Hospital Aachen in Germany have an official agreement for the transfer of patients; consequently, dozens of patients are transferred each year between the 2 hospitals. The same applies for the University Hospital Maastricht in the Netherlands and the General Hospital Vesalius in Belgium, between which nearly 100 patients are transferred each year. In an official publication of the European Commission (D. Byrne, Maastricht Conference on Cross-Border Health Care, Maastricht, the Netherlands, June 8, 2004), the EMR was mentioned as a model region for the European Union in the field of cross-border healthcare and cross-border cooperation of hospitals. Furthermore, in July 2008, establishment of a pan-European university hospital was announced, a collaboration among the university hospitals of Maastricht in the Netherlands and Aachen in Germany.

Of particular concern is cross-border dissemination of multidrug-resistant bacteria, such as methicillin-resistant *Staphylococcus aureus* (MRSA). The 3 countries forming the EMR differ considerably in the prevalence of hospital-isolated MRSA (23.6%, 13.8%, and 0.6% in Belgium, Germany, and the Netherlands, respectively) (*2*). Consequently, cross-border transfer of patients may affect the dissemination and prevalence of MRSA, particularly when patients are transferred from countries with a relatively high prevalence to a country with a low prevalence.

A study of MRSA isolates from the EMR between December 1999 and February 2004 showed that isolates from clonal complex (CC) 5 and CC 8, which harbor the resistance elements staphylococcal cassette chromosome *mec* (SCC*mec*) types I–IV, had been disseminated in the EMR (*2*). Our aim was to investigate the emergence, dissemination, and diversity of MRSA clones in the EMR during a 10-month period in 2005 and 2006 and to compare the results with those of the previous study. We used sequencing of the short sequence repeat (SSR) region of the *S. aureus* protein A gene (*spa* typing), multilocus sequence typing (MLST), and SCC*mec* typing by PCR to investigate the genetic background of all MRSA isolates. The *spa* locus was typed to provide more detailed information about prevalent MRSA clones in the EMR, especially because the previous study used only MLST analyses on a small subset of isolates (*2*). Finally, because an increase of Panton-Valentine leukocidin (PVL)–positive MRSA isolates in the Netherlands has recently been observed (*3*), we investigated the possible spread of PVL-positive MRSA clones into hospitals in the EMR, as well as the prevalence of the virulence factors collagen adhesion (CNA) and toxic shock syndrome toxin-1 (TSST-1).

## Materials and Methods

### MRSA Isolates

We investigated 257 MRSA isolates, cultured during July 2005–April 2006 from 8 geographically closely related hospitals in the EMR. The hospitals included 1 hospital in Belgium (General Hospital Vesalius, Tongeren, 355 beds), 2 hospitals in Germany (General Hospital Dūren, 521 beds, and Marien Hospital, Aachen, 321 beds), and 5 hospitals in the Netherlands (Atrium Medical Center, Heerlen, 811 beds; Orbis Medical and Care Center, Sittard, 578 beds; Laurentius Hospital, Roermond, a 458-bed general hospital; University Hospital Maastricht, a tertiary hospital, 680 beds; and VieCuri Medical Center, Venlo, a 554-bed general hospital). The 257 MRSA isolates comprised 44 from Belgium, 92 from Germany, and 121 from the Netherlands. Isolates from the Belgian and German hospitals were from patients with MRSA infection; Dutch isolates were from patients carrying MRSA who were admitted to the Dutch hospitals. All isolates were identified as *S. aureus* by Gram stain, catalase, and coagulase testing. The presence of the *mecA* gene was determined as described previously (*2*).

### Antimicrobial Drug Susceptibility Testing

The susceptibility pattern of the MRSA isolates was determined according to the guidelines of the Clinical and Laboratory Standards Institute (*4*). Susceptibility to the following antimicrobial agents was determined as MIC: cefaclor, cefuroxime, clindamycin, ciprofloxacin, clarithromycin, gentamicin, linezolid, moxifloxacin, oxacillin, penicillin, rifampin, teicoplanin, tetracycline, trimethoprim/sulfamethoxazole, and vancomycin. The susceptibility to fucidic acid and mupirocin (Rosco, Taastrup, Denmark) was determined by using the disk-diffusion method (*5**,**6*). MRSA isolates resistant to clarithromycin were tested for inducible clindamycin resistance by using the D-test (*7*).

### Typing Methods

SCC*mec* typing was performed as described by Oliveira et al. (*8*) with the modification described previously (*2*). SCC*mec* type I elements that lack locus A (*pls* region) are indistinguishable (*9*) from SCC*mec* type IV elements when the method of Oliveira et al. is used (*8*). Furthermore, locus D (*dcs* region) is detected in both SCC*mec* types IV and VI (*10*). Therefore, SCC*mec* elements that were typed as SCC*mec* type IV using the method of Oliveira et al. (*8*) were further analyzed for presence of the *ccrAB* gene. SCC*mec* elements that could not be typed with the method of Oliveira et al (*8*) were further analyzed by using the methods of Ito et al. (*11*) and Zhang et al. (*12*).

Real-time amplification of the *spa* gene was performed as described previously, followed by sequencing of the SSR region (*13*). The *spa* types were clustered into *spa*-CCs using the algorithm Based Upon Repeat Pattern (BURP) with the Ridom StaphType version 1.4 software package (www.ridom.de). Because *spa* typing, together with the algorithm BURP, yields results concordant with typing results obtained by MLST and pulsed-field gel electrophoresis (*13*), the associated CCs, as determined with MLST, were allocated through the Ridom SpaServer (http://spaserver.ridom.de). To confirm the association between MLST and *spa* typing, in combination with BURP, MLST was performed on a representative set of 12 strains of each major *spa* type and *spa*-CC (*2*).The presence of CNA, PVL, and TSST-1 was determined with real-time PCR assays (*14**,**15*).

## Results

### Antimicrobial Drug Susceptibility Patterns

All 257 MRSA isolates were resistant to the β-lactam antimicrobial agents cefaclor, cefuroxime, oxacillin, and penicillin and were susceptible to linezolid, teicoplanin, and vancomycin. Most isolates were also resistant to ciprofloxacin (84%) and moxifloxacin (82%). The Dutch MRSA isolates were more often susceptible to ciprofloxacin and moxifloxacin than were the Belgian and German isolates (Table 1) (p<0.05). Furthermore, 78% of the MRSA isolates were resistant to clarithromycin, and 62%, to clindamycin. Susceptibility for clarithromycin and clindamycin differed by country (Table 1). A total of 41 MRSA isolates (19 from Belgium, 5 from Germany, and 17 from the Netherlands) was resistant to clarithromycin and susceptible to clindamycin. The D-test showed that 31 (76%) of these 41 MRSA isolates had the inducible clindamycin resistant phenotype, including 15 from Belgium, 5 from Germany, and 11 from the Netherlands.

**Table 1:** Non–β-lactam antimicrobial drug resistance patterns of 257 MRSA isolates in the Euregio Meuse-Rhin region, July 2005–April 2006*

Country	No. isolates	No. (%) resistant MRSA isolates									
		CIP	MXF	CLI	GEN	CLR	SXT	TET	RIF	MUP	FUC
Belgium	44	44 (100)	43 (98)	5 (11)	2 (5)	24 (55)	0	4 (9)	0	4 (9)	0
Germany	92	89 (97)	89 (97)	78 (85)	5 (5)	83 (90)	0	3 (3)	2 (2)	1 (1)	1 (1)
The Netherlands	121	84 (69)	79 (65)	76 (63)	11 (9)	93 (77)	3 (2)	22 (18)	0	8 (7)	1 (1)
Total	257	217 (84)	211 (82)	159 (62)	18 (7)	200 (78)	3 (1)	29 (12)	2 (1)	13 (5)	2 (1)

*MRSA, methicillin-resistant *Staphylococcus aureus*; CIP, ciprofloxacin; MXF, moxifloxacin; CLI, clindamycin; GEN, gentamicin; CLR, clarithromycin; SXT, trimethoprim/sulfamethoxazole; TET, tetracycline; RIF, rifampin; MUP, mupirocin; FUC, fucidic acid. No isolates showed resistance to linezolid, vancomycin, or teicoplanin.

### Distribution of MRSA Clones

SCC*mec* type IV was predominant in MRSA isolates from Belgium (77%), whereas MRSA isolates from Germany harbored mainly SCC*mec* type II (82%). MRSA isolates from the Dutch region harbored both SCC*mec* type II and IV (27% and 65%, respectively). Although 25 (10%) of the 257 MRSA isolates harbored an SCC*mec* element that could not be typed with the method of Oliveira et al. (*8*), they could be typed with the other methods. Seven MRSA isolates from Belgium harbored a SCC*mec* type III element that lacked Tn*554*, which is usually characteristic for SCC*mec* type III. From the German region, 1 isolate that had a nontypeable SCC*mec* element harbored *ccrC*, locus E, and Tn*554*. The method of Zhang et al. (*12*) classified this element as SCC*mec* type III. In the Netherlands, 17 MRSA isolates contained a nontypeable SCC*mec* element as defined by Oliveira et al. (*8*). Ten of these were classified as SCC*mec* type IV, lacking locus D. The remaining 7 harbored *ccrC*, characteristic for SCC*mec* type V, and were classified as such with the method of Zhang et al. (*12*).

The 257 MRSA isolates were classified into 36 different *spa* types, and BURP analysis showed 6 *spa*-CCs, 4 singletons, and 3 *spa* types that were excluded from the analysis because the *spa* region was <5 *spa* repeats long (Table 2). MLST analyses showed 10 different STs among the 12 MRSA strains (Table 2). In the EMR, *spa*-CC 045 (MLST CC5; 21%) and *spa*-CC 038 (MLST CC45; 75%) were found predominantly among MRSA isolates from the Belgian region; *spa*-CC 045 (MLST CC5; 85%) was found among isolates from the German region. The Dutch MRSA isolates were grouped into *spa*-CC 045 (MLST CC5; 39%), *spa*-CC 019/012/318/011/108 (MLST CC30 and CC398; 15%), *spa*-CC 038 (MLST CC45; 15%), *spa*-CC with no founder 5 (MLST CC8; 16%), and *spa*-CC with no founder 6 (MLST CC 45; 5%).

**Table 2:** Composition of the *spa*-CCs of 257 MRSA isolates in the Euregio Meuse-Rhin region, July 2005–April 2006*

*spa*-CC	No. (%) isolates	No. (%) *spa* types	*spa* types	ST	CC
045	134 (52)	9 (25)	t002, **t003**, t041, **t045**, t179, **t447**, t504, t838, t1107	ST5/ST225†	CC5
019/012/318/011/108	19 (7)	7 (19)	**t011**, **t012**, t019, t034, t108, t318, t582	ST36/ST398‡	CC30/CC398
038	58 (22)	5 (14)	**t038**, t161, **t740**, t1288, t1310	ST45	CC45
044/042	4 (2)	4 (11)	t042, **t044**, t131, **t345**	ST728/ST772§	CC1/CC80
No founder 5	22 (9)	2 (6)	**t008**, t622	ST8	CC8
No founder 6	8 (3)	2 (6)	**t040**, t553	ST45	CC45
Singletons	8 (3)	4 (11)	**t223**, t375, t682, t786	ST217¶	CC22/CC89
Excluded#	4 (2)	3 (8)	t457, t779, t1011		
Total	257 (100)	36 (100)			

*CC, clonal complex; MRSA, methicillin-resistant *Staphylococcus aureus*; ST, sequence type. **Boldface** indicates *spa* types on which multilocus sequence typing analysis was performed. †The strains *spa* typed as t003 and t045 had ST225, a single-locus variant of ST5 at the *tpi* locus. The strain *spa* typed as t447 had ST5. ‡The strain *spa* typed as t011 had ST398, and the strain *spa* typed as t012 had ST36. §The strain *spa* typed as t044 had ST728, a single-locus variant of ST80 at the *tpi* locus. The strain *spa* typed as t345 had ST772, a single-locus variant of ST1 at the *pta* locus. ¶The strain *spa* typed as t223 had ST217, a single-locus variant of ST22 at the *tpi* locus. #*spa* types with <5 *spa* repeat.

The ST5-MRSA-II (New York/Japan) clone was found mainly in Germany and the Netherlands, and the ST45-MRSA-IV (Berlin) clone was found in Belgium and the Netherlands. Furthermore, the ST5-MRSA-IV (Pediatric) clone was found among the Dutch isolates. The MRSA isolates classified as CC30 (ST30-MRSA-IV and ST36-MRSA-II) were found only in the Netherlands. Most of the ST8-MRSA-IV (UK EMRSA-2/6) isolates were found in the Netherlands. Furthermore, several ST398-MRSA-IV and ST398-MRSA-V isolates were found in certain Dutch hospitals (Figure, Table 3).

**Figure:**
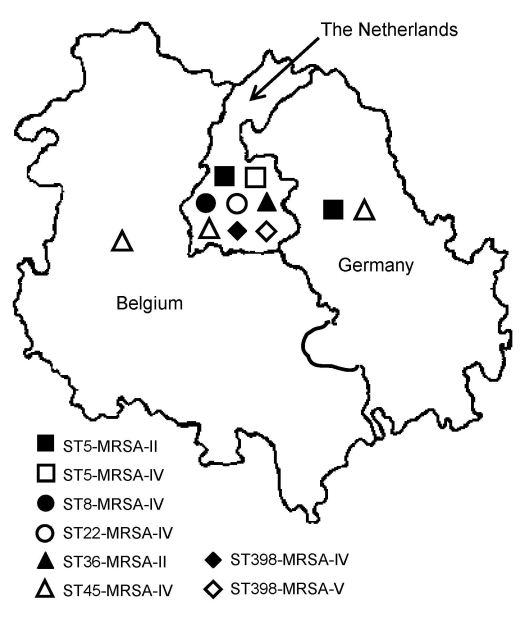
Distribution of the major methicillin-resistant *Staphylococcus aureus* (MRSA) clones in the Euregio Meuse-Rhin region, July 2005–April 2006.

**Table 3:** Distribution of MRSA clones in the Euregio Meuse-Rhin region, by country, July 2005–April 2006*

MRSA clone	No. isolates			
	Belgium	Germany	The Netherlands	Total
ST1-MRSA-V			1	1
ST5-MRSA-I	1	1	1	2
ST5-MRSA-II	1	75	30	106
ST5-MRSA-IV		2	17	19
ST8-MRSA-IV	1	2	19	22
ST22-MRSA-IV			4	4
ST30-MRSA-IV			3	3
ST36-MRSA-II			3	3
ST45-MRSA-IV	33	8	25	66
ST80-MRSA-IV		1	2	3
ST89-MRSA-I			1	1
ST89-MRSA-V			1	1
ST398-MRSA-IV			7	7
ST398-MRSA-V			5	5
NT MRSA	7†	1‡	2§	17
Excluded¶	1	2	1	4
Total	44	92	121	257

*MRSA, methicillin-resistant *Staphylococcus aureus*; ST, sequence type; NT, nontypeable. Based on *spa* and staphylococcus cassette chromosome (SCC) *mec* typing. †These strains were classified into clonal complex (CC) 5 and had a nontypeable SCC*mec* type III element. ‡This strain had a nontypeable SCC*mec* element belonging to CC30. §These strains harbored SCC*mec* type IV and could not be classified into a CC. ¶*spa* types with <5 *spa* repeats.

### Prevalence of Virulence Factors

Eleven (5%) of the 257 MRSA isolates were PVL-positive. These isolates were associated with different genetic backgrounds, i.e., ST1-MRSA-V (1 Dutch isolate), ST8-MRSA-IV, ST30-MRSA-IV (2 Dutch isolates each), ST45-MRSA-IV (1 isolate from Germany), ST80-MRSA-IV (1 isolate from Germany and 2 from the Netherlands), ST89-MRSA-IV and ST89-MRSA-V (1 Dutch isolate each). Six of the PVL-positive isolates were positive for the *cna* gene, and none harbored the *tst* gene.

Nine (4%) of the 257 MRSA isolates were positive for the *tst* gene, 4 isolates were classified as ST22-MRSA-IV, 3 as ST36-MRSA-II, 1 as ST30-MRSA-IV, and 1 could not be classified as an MRSA clone (*spa* type t779). All isolates were from the Netherlands and were positive for the *cna* gene; none harbored PVL.

Ninety-five (37%) of the 257 MRSA isolates were positive for the *cna* gene (34 from Belgium, 9 from Germany, and 52 from the Netherlands). All MRSA isolates from CC30, CC45, and ST398 harbored the *cna* gene. Furthermore, 1 isolate from CC5, 1 from CC80, 6 classified as singletons (associated with ST22 and ST89), and 2 excluded from the BURP analyses were positive for the *cna* gene.

## Discussion

Because cross-border healthcare is an issue in the EMR, and the prevalence of MRSA differs among the countries forming the EMR, studying the possible emergence, spread, and diversity of MRSA clones within and among these countries is important (*2*). In addition to MRSA clones from CC5 and CC8, found previously in the EMR, we observed MRSA isolates from CC30 and CC45. Furthermore, the Dutch isolates had a more heterogeneous genetic background than did MRSA isolates from Belgium and Germany. The prevalence of PVL-positive MRSA isolates, belonging to ST1, 8, 30, 80 and 89, was higher than that found in the previous study (5% vs. 1.3%) (*2*).

The antimicrobial susceptibility of the MRSA isolates depends on the *S. aureus* lineage. The observation that the Dutch MRSA isolates were more often susceptible to ciprofloxacin and moxifloxacin than were isolates from Belgium and Germany can be explained by the fact that the isolates associated with ST5-MRSA-IV, ST22-MRSA-IV, and ST30-MRSA-IV, which were susceptible to ciprofloxacin and moxifloxacin, were mainly observed in the Netherlands. Although ST22-MRSA-IV is commonly susceptible to tetracycline, the ST22-MRSA-IV isolates in this study were resistant to tetracycline (*16*). *S. aureus* can harbor resistance genes on mobile genetic elements on the genome, such as Tn*554*, as well as on plasmids, and these can be exchanged among *S. aureus* lineages, possibly because of antimicrobial drug pressure (*17*).

Primarily because of the Dutch “search-and-destroy” policy, isolates derived from colonized persons were available from the Netherlands, whereas isolates from Belgium and Germany were derived from infections. However, nasal carriers are at increased risk of acquiring MRSA infection (*18*). Consequently, not preventing the spread of MRSA among nasal carriers could lead to MRSA infection among these persons. Furthermore, the molecular epidemiology of MRSA can vary widely among hospitals. In the Dutch hospitals of the EMR, MRSA clones in each hospital were diverse, whereas in the Belgian hospital and 2 German hospitals, 1 MRSA clone predominated, showing that the number of hospitals is unlikely to have biased the results of our study.

Most of the MRSA isolates from Belgium were associated with the Berlin clone (ST45-MRSA-IV). This clone has previously been found in Belgium, Germany, and the Netherlands (*19*). Most of the MRSA isolates from Germany were associated with the New York/Japan clone (ST5-MRSA-II), previously found in Belgium and Germany (*2**,**19*). Most of the Dutch MRSA isolates belonged to 5 MRSA clones (Table 3). Twenty-five percent of the Dutch isolates were associated with the New York/Japan clone (ST5-MRSA-II), which has not been previously found in the Netherlands. The Pediatric clone (ST5-MRSA-IV), which represented 14% of the Dutch isolates, has been found in Belgium but not in the Netherlands (*20**,**21*). The Berlin clone (ST45-MRSA-IV), comprising 21% of the Dutch isolates, and the UK EMRSA-2/-6 clone (ST8-MRSA-IV), comprising 16% of the Dutch isolates, have been described in all 3 EMR countries (*19**,**20*). In addition, some less prevalent MRSA clones were observed. Four *tst*-positive MRSA isolates were associated with the UK EMRSA-15 clone (ST22-MRSA-IV), previously found in Belgium and Germany but not in the Netherlands (*19**,**20*). Three Dutch MRSA isolates (*spa* type t012), harboring SCC*mec* type II, were associated with the CC30 lineage. These isolates might be derived from the UK EMRSA-16 (ST36-MRSA-II) clone (*spa* type t018) because *spa* types t012 and t018 differ in 1 *spa* repeat (r24) and are thus related. Furthermore, both clones harbor the *cna* and *tst* genes (*22**,**23*). The highly endemic UK EMRSA-16 clone has not been observed before in the Netherlands, although this clone has previously been found in Belgium (*24*). Seven and 5 of the Dutch MRSA isolates were associated with ST398-MRSA-IV and ST398-MRSA-V, respectively, MRSA clones usually observed in pigs and among screening samples from pig farmers (*25*). The ST398 clone is now observed among screening samples of veterinarians from many countries in Europe, including Belgium and Germany (*26*). However, ST398 also has been isolated from several forms of human infections in Germany (*27*). The ST398 isolates from our study were positive for the *cna* gene, suggesting a higher virulence than that of the CNA-negative German ST398 MRSA isolates (*27*). One Dutch MRSA strain was associated with the ST30-MRSA-IV clone, previously reported in Belgium, Germany, and France (*20**,**21**,**28*). The more diverse genetic background among MRSA isolates in the Dutch part of the EMR and the close cooperation of hospitals in the EMR might suggest that importation of MRSA from Belgium and Germany has occurred through cross-border healthcare (Table 4) (*2*). Other, less likely, explanations for the diversity of MRSA clones in the Netherlands are the spread of MRSA from countries other than Belgium or Germany (*19*) and the emergence of new MRSA clones in vivo through transfer of the SCC*mec* element from methicillin-resistant coagulase-negative staphylococci to methicillin-sensitive *S. aureus* strains (*29*).

**Table 4:** Suggested cross-border dissemination of the major MRSA clones in the Euregio Meuse-Rhin region, July 2005–April 2006*

MRSA clone	Previously observed in/possible dissemination from
ST5-MRSA-II	Belgium, Germany
ST5-MRSA-IV	Belgium
ST8-MRSA-IV	Belgium, Germany, the Netherlands
ST22-MRSA-IV	Belgium, Germany
ST30-MRSA-IV	Belgium, Germany
ST36-MRSA-II	Belgium
ST45-MRSA-IV	Belgium, Germany, the Netherlands

*MRSA, methicillin-resistant *Staphylococcus aureus*; ST, sequence type.

We could not determine the SCC*mec* type for 10% of the MRSA isolates by using the method of Oliveira et al. (*8*). This percentage was similar to that found in other studies (*30**,**31*) but higher than the 3% previously found in the EMR (*2*). The relatively large number of nontypeable SCC*mec* types found in this study, probably formed by homologous recombination among SCC*mec* elements, supports the need for a new system for SCC*mec* typing and nomenclature (*19*).

The 7 Belgian MRSA isolates with the nontypeable SCC*mec* type III element were associated with CC5 and had the related *spa* types t045 and t1107 (http://spaserver.ridom.de). Although SCC*mec* type III usually is found in the CC8 genetic background, such as in the ST239-MRSA-III clone, an MRSA associated with CC5 (*spa* type t045) and harboring SCC*mec* type III recently was observed in Belgium (*32*). This might suggest that a new MRSA clone, ST5-MRSA-III, is beginning to emerge in Belgium.

The nontypeable SCC*mec* element of the German MRSA isolate harbored locus E and *ccr*C, specific for SCC*mec* type V (*2*), and Tn*554*, normally carried by SCC*mec* type II, III, and SCCmercury. Zhang et al. (*12*) classified this element as SCC*mec* type III, but the SCC*mec* type III-specific primers used by this method are situated near locus E on SCCmercury (*33*), indicating that this element could be a SCCmercury element containing *mecA*. Further investigation is needed into the structure of this element.

Previous studies have shown that MRSA isolates classified as community-associated usually harbor either SCC*mec* type IV or V, and often PVL, but may differ in their genetic backgrounds (CC1, CC8, CC30, CC59 and CC80) (*34*). In the EMR, 5% of the MRSA isolates were positive for PVL, which is higher than the previously reported 1.3% (*2*). Thus, PVL-positive MRSA isolates with a heterogeneous genetic background are emerging in the EMR.

PVL-positive MRSA isolates associated with ST8-MRSA-IV, ST30-MRSA-IV, and ST80-MRSA-IV have been isolated in the Netherlands (*3**,**35*). In the present study, 2 of the PVL-positive MRSA isolates harbored SCC*mec* type V. The different genetic background of these isolates, i.e., ST89 and ST772, a single-locus variant of ST1 at the *pta* locus, might suggest that SCC*mec* type V was introduced on different occasions into different *S. aureus* lineages. A PVL-positive ST772-MRSA-V has been observed in Germany (*36*). One of the PVL-positive isolates harbored SCC*mec* type I, and such isolates with a ST30 and ST37 genetic background have been described in the Netherlands (*3*). Although a recent study suggested that CNA and PVL combined contribute to virulence, only 6 of the 11 PVL-positive MRSA isolates from the EMR harbored the *cna* gene (*37*). Further studies are needed to investigate the contribution of the combination of CNA and PVL to virulence.

The genetic background of 1 PVL-positive ST45-MRSA-IV isolate from Belgium was similar to that of the Berlin clone. Hitherto, only PVL-negative isolates with this background have been found in EMR countries (*19**,**20*). PVL-positive MRSA isolates, associated with the major CA-MRSA clones (ST8-MRSA-IV, ST30-MRSA-IV, and ST80-MRSA-IV) have been reported from Belgium (*38*). Because PVL is situated on a phage, the genes encoding PVL might have been transferred to *S. aureus* with a CC45 genetic background (*34*).

Our study found a PVL-positive MRSA isolate from Germany with *spa* type t042 (*spa* repeat pattern r26r23r12r34r34r33r34). This *spa* type is strongly related to *spa* types t044 and t131 (*spa* repeat patterns r07r23r12r34r34r33r34 and r07r23r12r34r33r34, respectively), which are usually associated with the CA-MRSA ST80-MRSA-IV clone found in Germany (*34*).

The *cna* gene has been previously observed among MRSA isolates from CC22, CC30, and CC45 (*23**,**29*). Therefore, the presence of the *can* gene might, together with *spa* typing, be used as a marker for different genetic backgrounds.

MRSA clones associated with the hospital associated-MRSA CCs 5, 8, 22, 30, and 45, the PVL-positive CA-MRSA CCs 1, 8, 30, 80, and 89, as well as MRSA related to pigs (ST389-MRSA-IV/V) were observed in the EMR. Dissemination of these clones is possible because of the introduction of new MRSA clones associated with travel; with patients who have previously been admitted to a hospital abroad (cross-border healthcare); or with other high-risk patients, such as pig-farmers and their families. Therefore, a cross-border search-and-contain policy may help control the further spread of MRSA and reduce the financial cost to hospitals, nursing homes, and the community in the EMR.
